# Improved Visual Inspection through 3D Image Reconstruction of Defects Based on the Photometric Stereo Technique

**DOI:** 10.3390/s19224970

**Published:** 2019-11-14

**Authors:** Sanao Huang, Ke Xu, Ming Li, Mingren Wu

**Affiliations:** 1 University of Science and Technology Beijing, Collaborative Innovation Center of Steel Technology, Beijing 100083, China; huangsanao@cgnpc.com.cn (S.H.); mingrw_exc@163.com (M.W.); 2 In-Service Testing Center, CGN Inspection Technology Co., Ltd, Suzhou 215000, China; liming@cgnpc.com.cn

**Keywords:** nuclear power plant, visual inspection, photometric stereo, 3D reconstruction

## Abstract

Visual inspections of nuclear power plant (NPP) reactors are important for understanding current NPP conditions. Unfortunately, the existing visual inspection methods only provide limited two-dimensional (2D) information due to a loss of depth information, which can lead to errors identifying defects. However, the high cost of developing new equipment can be avoided by using advanced data processing technology with existing equipment. In this study, a three-dimensional (3D) photometric stereo (PS) reconstruction technique is introduced to recover the lost depth information in NPP images. The system uses conventional inspection equipment, equipped with a camera and four light-emitting diodes (LEDs). The 3D data of the object surface are obtained by capturing images under multiple light sources oriented in different directions. The proposed method estimates the light directions and intensities for various image pixels in order to reduce the limitation of light calibration, which results in improved performance. This novel technique is employed to test specimens with various defects under laboratory conditions, revealing promising results. This study provides a new visual inspection method for NPP reactors.

## 1. Introduction

The reactor pressure vessel (RPV) of a nuclear power plant (NPP) requires periodic inspection to ascertain current conditions. Any defects on the internal surfaces may undermine the safe operation of the NPP. Moreover, exposure to irradiation and corrosive coolants, or damage caused by manufacturing and outage activities, could accelerate the growth of these defects [1]. Thus, inspection systems and implementation practices must be capable of detecting small flaws, to prevent them from growing to a size that could compromise the leak tightness of the pressure boundary.

Visual inspection is the main method for detecting defects, structural integrity issues, or leakage traces on the surface of key components in an NPP. Owing to its advantages, the demand for more advanced visual inspection techniques is increasing. The U.S. Nuclear Regulatory Commission (NRC) has approved the use of high-resolution cameras for inspecting specific areas of key NPP components instead of ultrasonic examination [2]. In addition, machine vision technology has been applied to measure fuel assembly deformation [3].

Visual inspection systems capture images of the surfaces of objects by using an image sensor, a charge-coupled device (CCD), or a complementary metal-oxide semiconductor (CMOS), with appropriate optical tools and lighting conditions. The visual module is typically composed of light sources and image sensing units, and completes the inspection with the aid of automated tools. Companies such as AREVA and CYBERIA in France, DEKRA in Germany, Ahlberg in Sweden, DIAKNOT in Russia, and Westinghouse in the United States have been actively developing such devices. The majority of inspection systems are equipped with high-definition cameras, include a choice of light sources, from halogen to light-emitting diode (LED) lights, and boast anti-irradiation and waterproof properties. The types of automated tools are diverse. Some products have data processing functions, which are becoming increasingly popular. For example, AREVA’s latest RPV device, SUSI 420 HD, is equipped with a high-definition camera and four adjustable high-power LEDs, but the sizing of indications is limited to the length measurement [4].

Existing NPP visual inspection methods still use two-dimensional (2D) images to identify defects. Occasionally, the lack of three-dimensional (3D) observations makes it difficult to evaluate certain observations—specifically, the potential size of the defect. Some inspection tasks can only be performed using 3D analysis methods, because surface defects may only appear with changes in the shape of the surface [5]. Therefore, visual inspections can be improved by detecting changes in the 3D surface. Three-dimensional shape reconstruction methods based on visible light include structured light and stereo vision technology. For example, the laser 3D scanner of the Newton Laboratory can map NPP fuels and check the size of defects [6]. Karl Storz laser technology, called MULTIPOINT, is a 3D laser system with 49 laser points that enables cooperation between the camera and the software to detect the surface structure of the subject [7]. However, few of these devices can operate individually without additional overheads. Therefore, to save time and money, it is preferable to use conventional devices to extract and analyze 3D data for defects. One such method achieves 3D reconstruction of the inner surfaces of boreholes or cavities using conventional endoscopy equipment [5]. However, this method does not provide system calibration results or evaluation criteria of the results; thus, further improvement is required. Furthermore, the 3D visualization function obtained through the shape from motion (SFM) method can be used to inspect the advanced, air-cooled core in an NPP, but a lack of surface features limits the application of this method [8].

The photometric stereo (PS) technique recovers 3D shapes from multiple images of the same object, taken under different illumination conditions. As a result of the pioneering work of Woodham, it has been widely applied to 3D surface reconstruction [9]. This technique features two advantages: low hardware costs and low computation costs. In the field of industrial inspection, PS has improved the detection of very small surface defects [10,11]. The Lambertian model assumption is commonly used where albedo is assumed to be constant. Although this does not necessarily correspond to the actual conditions, there are approaches available to realize the normal calculations [12,13,14]. However, for models with non-Lambertian reflection properties, highlight and shadow processing requires additional images [13].

The assumption of the light source and the demand for extensive calibration procedures in conventional PS limit its applicability [15]. Some previous studies have established illumination models that conform to actual conditions, such as near-field light models [16,17,18,19]. Light calibration, which aims to estimate the light direction and intensity, often requires a specific equipment or a dedicated process [20]. However, equipment such as precise calibration spheres or positioning devices are unlikely to be available in actual applications. Some studies have proposed fully uncalibrated or semi-calibrated PS methods. A fully uncalibrated, near-light PS method achieves the calculation of the light positions, light intensities, normal, depth, and albedo without making any assumptions about the geometry, lights, or reflectance of the scene [21]. Regarding semi-calibrated PS, various approaches achieve light intensity calibration [22]. However, additional information is required to solve the high-dimensional ambiguity, and more importantly, at least 10 images are typically required [15]. To apply PS to an NPP environment, a fully automatic calibration method should be designed.

This study employs the PS method with a conventional NPP visual inspection device to reconstruct the 3D shapes of defects from visual inspection images. Additional contributions include the development of an auto-calibrated, near-field light calibration method that can easily and accurately calibrate the light source to meet the demands of practical applications. Moreover, depth information can be extracted from the captured images, which enables better and more reliable visualization of surface defects.

The structure of this paper is as follows: The PS formulation is briefly introduced in Section 2. The algorithm details for extracting 3D information from captured images are described in Section 3, as well as the method for estimating light direction and intensity. The experimental setup and results are discussed in Section 4, and the conclusions are presented in Section 5.

## 2. Photometric Stereo Technique

PS techniques use multiple images taken from the same viewpoint, but under illumination from different directions, in order to recover the surface orientation from a known combination of reflectance and lighting values. The depth and shape of the surface can be obtained via the reconstruction algorithms. The objects in the scene are Lambertian, and the illumination is a distant point light; the measured image intensity at a point $P\left(x,y,z\right)$ can be written as:

(1) $$I=\rho \langle l,n\rangle E$$

where *ρ* is the albedo at *P*; ***l*** is the light direction; ***n*** is the surface normal; and *E* is the light irradiance. The image intensity *I* can be measured per-pixel. 

At least three independent light sources are required.

Suppose we have *M ≥* 3 images under varying light directions, which we denote as direction vectors ***l_1_, ….., l_M_*** $\in {\mathbb{R}}^{3}$. By assuming equal light irradiance; i.e., $\rho E_{}=\rho E_{1}=\cdots \cdots =\rho E_{M}$, we can estimate the normal vector ***n*** on a surface point *P* by solving the pseudo-inverse matrix of ***L*:**

(2) $$n=\frac{{\left(L^{T}L\right)}^{-1}L^{T}I}{\left\|{\left(L^{T}L\right)}^{-1}L^{T}I\right\|}$$

where $L=\left[\begin{array}{c} l_{1}\\ \vdots \\ l_{M} \end{array}\right],I={\left[I_{1},\cdots ,I_{M}\right]}^{T}$.

The surface normal can be computed using Equations (1) and (2). Then, the recovery of the surface from the computed normal can be achieved using algorithms, including optimization iterative methods and pyramid reconstruction algorithms. The entire procedure of PS-based 3D shape reconstruction includes: calibration, surface normal computation, and shape reconstruction from the normal.

## 3. Three-Dimensional Shape Reconstruction of Defects

### 3.1. Existing Two-Dimensional Image Capture and Data Analysis Method

The RPV generally consists of a cylindrical part, a spherical part, and nozzles. Although its size varies, the diameter of the cylinder is always approximately 4000 mm. An examination of the entire internal surface of the reactors is required to detect surface disorders, deformation, or other important defects; i.e., (1) mechanical defects, such as scratches or impact damages caused by foreign bodies; (2) metallurgical defects, such as cracks or arc strikes; and (3) corrosion pitting or deposits. Moreover, the defects may need to be evaluated both qualitatively and quantitatively.

During the inspection process, tools are used to position the cameras close to the different areas requiring inspection. Scanning is performed using tools to record videos of the internal surface, using cameras facing the wall within a field limited to the zone under examination. Technicians observe the video and images throughout the entire process to identify defects. If an abnormality or suspicious observation is recorded, the movement of the tool can be paused to allow more detailed information to be observed manually.

Because of its beneficial features, a pan/tilt/zoom (PTZ) setup, which normally consists of a camera, a number of surrounding light sources, lasers (optional), and a built-in pan/tilt unit, has been widely used as the visual module. The pan and tilt functions allow adjustment of the camera position to capture better images. Data analysis is performed by inspectors; therefore, suitable frames are required for observation and evaluation, along with other information recorded during the inspection, to make a qualified evaluation [23]. As the existing analysis method is based on 2D images, it is often difficult to evaluate defects due to a lack of depth information; thus, some inspection tasks cannot be solved using 2D analysis methods.

### 3.2. Photometric Stereo System

**Figure 1:**
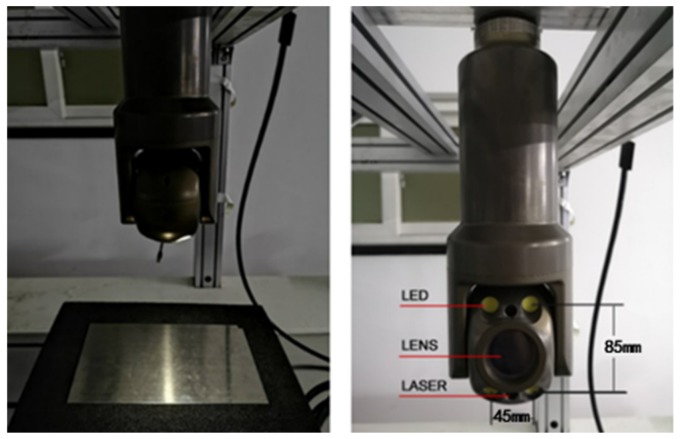
Pan/tilt/zoom (PTZ) image capture setup and light-emitting diode (LED) light layout dimensions (mm).

**Figure 2:**
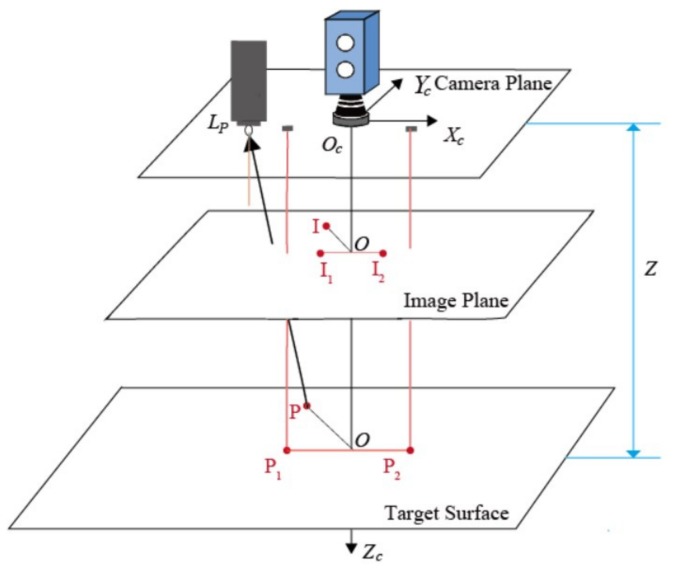
Diagram of the photometric stereo system.

PTZ is one type of module used for NPP visual inspection. In our configuration, it is equipped with four 30 W LED lights placed around a CMOS camera with 1920 × 1080 picture elements, attached with an F1.6~F3.0 lens, as shown in Figure 1. The LEDs exhibit approximately the same performance in terms of the radiant flux value and emitting angle. In the PTZ setup, all LED lamps are fixed to make their optical axes parallel to the viewing angle of the camera. Each LED can be dimmed in increments from 0% to 100%. In addition, two laser generators display reference points for length measurement; the distance between them is calibrated prior to the experiment. Practical inspections must be conducted at a viewing angle as perpendicular to the target surface as possible. As shown in Figure 2, *X_c_*, *Y_c_*, and *Z_c_* are the three axes of the camera coordinate system, which also represent the global coordinate system in our method. 

The first stage of the shape reconstruction algorithm—i.e., the calibration of camera parameters—begins by calibrating the camera intrinsic parameters using Toolbox in Matlab. Then, images are captured using the camera with the laser points turned on. Given these images, the following steps are taken:

(1) Estimate the distance between the PTZ (camera plane) and the target surface by using laser points and prior knowledge;
(2) Estimate ***l*** and *E* for each pixel by determining the relationship between each image pixel and its corresponding point on the target surface, using the near-field light model;
(3) Compute the normal by resolving the irradiance equations;
(4) Compute the final 3D surface shape from the normal field via an optimization algorithm.

The PS formulation for our configuration is briefly introduced in Section 2, and details of the algorithm are provided in Section 3.3 and Section 3.4.

### 3.3. Estimation of Light Direction and Intensity

Previous studies typically assume that the light sources are infinitely far from the surface, and generally adopt the parallel ray model. However, our system uses LEDs, which are very close to the target surface; thus, the lighting direction and intensity differ among various image areas. Moreover, the camera and lights are not fixed during an actual inspection. As previous light calibration methods are neither practical nor accessible for our application, it is necessary to design a fully automatic calibration method to estimate the light direction and intensity at each point.

In our configuration, the viewing angle is as perpendicular to the target surface as possible. In addition, the target surface is approximated as a planar surface that is assumed to be parallel to the image plane. Furthermore, the LED chip center and camera lens lie on a single plane, which is also parallel to the image plane. Accordingly, there are three parallel planes in the configuration, as shown in Figure 2.

The lighting direction for each point is decided by the position of the light *L_p_* and the point *P* on the target surface. Supposing that the camera plane is the horizontal plane in the coordinate system, *L_p_* can be determined by the PTZ structure. To determine the coordinate of point, the mapping relationship between image pixels and surface points must be determined, as well as the distance between the camera plane and the target plane. Thus, a two-stage process is designed:

(1) The distance *z* between the camera plane and the target plane is determined via using lasers;
(2) The orthographic projection-based method is used to determine how a point on the target surface is related to a pixel on the image plane.

Figure 2 shows that the viewing angle is aligned with the negative *z*-axis of the coordinate system, which simplifies the geometry calculation. The laser generators are fixed on PTZ, and their relative positions are known. Thus, $P_{1}\left(x_{1},y_{1},z\right)$ and $P_{2}\left(x_{2},y_{2},z\right)$, the intersections of the laser optical axis and the target plane, are also fixed. Their corresponding image pixels are $I_{1}\left(x_{1}^{\prime },y_{1}^{\prime },z_{}^{\prime }\right)$ and $I_{2}\left(x_{2}^{\prime },y_{2}^{\prime },z_{}^{\prime }\right)$, respectively, as shown in Figure 2. 

The assumption of orthographic projection has typically been used in the conventional PS, although the perspective projection has been demonstrated to be more realistic [24]. However, when the change in scene depth is small relative to the distance from the scene to the camera, an orthographic projection can be used instead of a perspective projection [25]. In our method, the viewing angle is kept as perpendicular to the target surface as possible, and the distance between the target surface and the camera is considerably greater than the depth of the defects. Therefore, it is reasonable to apply an orthographic projection model without resulting in a large deviation. 

The image magnification can be expressed as follows:

(3) $$m=\frac{∥P_{1}-P_{2}∥}{∥I_{1}-I_{2}∥}=\frac{f}{z}$$

where *m* is the imaging magnification, *f* is the camera focal length, $\left\|●\right\|$ denotes the length of a vector, and *z* is the distance between the target surface and the lens aperture, which is approximately equal to the distance between the surface plane and the camera plane. We denote *z*_0_ as the calibrated distance of reference $I_{1}\left(x_{1}^{},y_{1}^{},z\right)$ and $I_{2}\left(x_{2}^{},y_{2}^{},z\right)$; *z* can then be calculated using

(4) $$z=\frac{{\left[{\left(x_{2}^{}-x_{1}^{}\right)}^{2}+{\left(y_{2}^{}-y_{1}^{}\right)}^{2}\right]}^{1/2}}{{\left[{\left(x_{2}^{\prime }-x_{1}^{\prime }\right)}^{2}+{\left(y_{2}^{\prime }-y_{1}^{\prime }\right)}^{2}\right]}^{1/2}}z_{0}.$$

**Figure 3:**
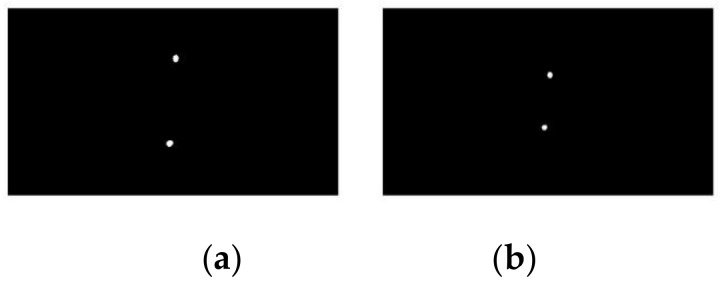
Laser points with different distances between the camera plane and the target plane: (**a**) 200 mm and (**b**) 350 mm.

Laser points with different distances between the camera plane and the target plane are shown in Figure 3. Thus, the next step is to define how a point on the target surface is related to a pixel on the image plane. 

As illustrated in Figure 2, there is a clear relationship between image pixels and their corresponding points. For any image pixel $I\left(x_{i}^{\prime },y_{i}^{\prime },z^{\prime }\right)$, the position of its corresponding point in the target surface can be defined as $P\left(kx_{i}^{\prime },ky_{i}^{\prime },z\right)$, where k=1/m. Therefore, the coordinate of *P* is decided by *k*, which can be determined by the laser points and *z*. The position of the LED,$L_{P}\left(x_{LED}^{},y_{LED}^{},0\right)$, can also be estimated from prior knowledge. Therefore, the light direction ***l***, or $\vec{LP}$, can be expressed as $\left(kx_{i}^{\prime }-x_{LED},ky_{i}^{\prime }-y_{LED},z\right)$.

For LED lights, the irradiance (*E*) on the target surface can be expressed as follows:

(5) $$E=\frac{I_{LED}\left(\theta \right)\cos\left(\theta \right)}{r^{2}}$$

where *θ* is the emitting angle of the LED, *I_LED_* denotes the radiant intensity of the LED, and *r* is the distance from the light source to the target point [17]. By transforming the parameters, *E* can also be calculated from Equation (5). Then, ***l*** and *E* can be determined for various image pixels $I\left(x_{i}^{\prime },y_{i}^{\prime }\right)$.

The goal of this step is to determine the lighting direction and intensity for each image pixel, which will improve the accuracy of the surface normal calculation, as well as the final 3D data quality.

### 3.4. Three-Dimensional Reconstruction using Photometric Stereo Technique

The PS procedure, assuming a Lambertian reflectance model, is applied for 3D shape reconstruction. The normal *n* is determined by using at least three images with various lighting conditions. In this study, they are calculated using the illustrated lighting direction and intensity estimation methods. Then, the image intensity follows inverse squared law, as

(6) $$I=\rho \frac{\left(n\times l\right)}{r^{2}}=\rho \frac{n\times \left(P-L_{p}\right)}{∥P-L_{p}{∥}^{3/2}}$$

where *r* is the distance between the light source and the surface point [17]. 

The normal, *n*, can be calculated using at least three equations; once it is solved, the surface shape can be reconstructed via the height estimation by a global iterative method [26,27].

Suppose each image has *W* rows that are indexed by *j*, and has *H* columns that are indexed by *i*. The pixel is therefore denoted as $\left(j,i\right)$, assuming its depth is $z\left(j,i\right)$, and the gradients in the *x* and *y* directions can be expressed as

(7) p=∂z(j,i)/∂i, q=∂z(j,i)/∂j

Then,

(8) $$n/\left\|n\right\|={\left(p,q,\left.-1\right)\right.}^{T}.$$

The image size is W×H, so that the discrimination function in the iterative method is

(9) $$E=\frac{1}{W\times H}\iint {\left(\frac{\partial z(j,i)}{\partial i}-p(j,i)\right)}^{2}+{\left(\frac{\partial z(j,i)}{\partial j}-q(j,i)\right)}^{2}didj.$$

## 4. Experimental Results and Discussion

Existing NPP visual inspection methods are not highly reliable for identifying small defects. This could be improved by using a camera with a higher resolution, which would produce a higher contrast between the defect and the metal surface [1]. However, it can also be difficult to discriminate between true and false defects; therefore, we tested specimens exhibiting small, hard to discern defects.

For the experiments, we used PTZ, introduced in Section 3.2. The layout dimensions of the lens and lights and the image capture setup is shown in Figure 1. With a beam fixing the PTZ, the camera looks downward, and the sample images are captured from the bottom. The four light sources, lasers, and lens are controlled via a controller. In our experiments, the defects were placed immediately below the camera. 

Firstly, to show the efficacy of the proposed method for estimating *z*, the estimated distances were compared with the set values, as listed in Table 1, using a distance of 253 mm as the calibration point. The method provides accurate results, ranging from 133–493 mm. The errors were predominantly due to the orthographic image formation model, which does not always precisely reflect the actual conditions. These deviations are also related to the accuracy of laser point extraction.

**Figure 4:**
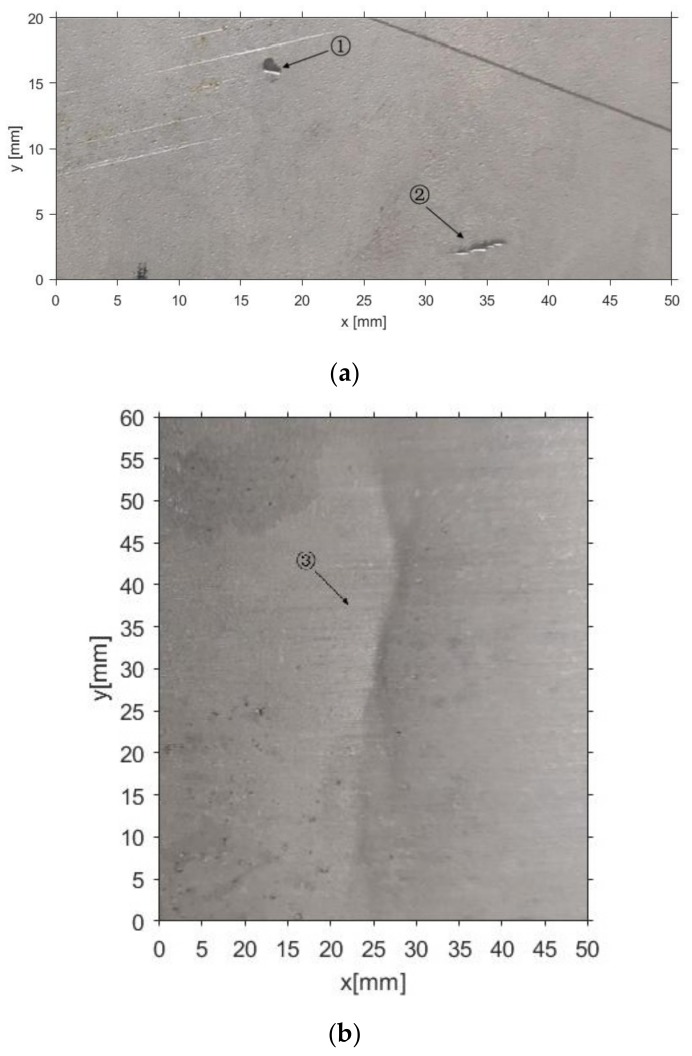
Images of test specimens: (**a**) 50 × 20 mm containing two dents (defects 1 and 2); (**b**) size 50 × 60 mm, containing one pit (defect 3).

The remaining experiments were conducted with two metal plates, as shown in Figure 4. One contains two small dents, labelled as defect 1 and defect 2 respectively, and the other one contains a pit, labelled as defect 3.

**Figure 5:**
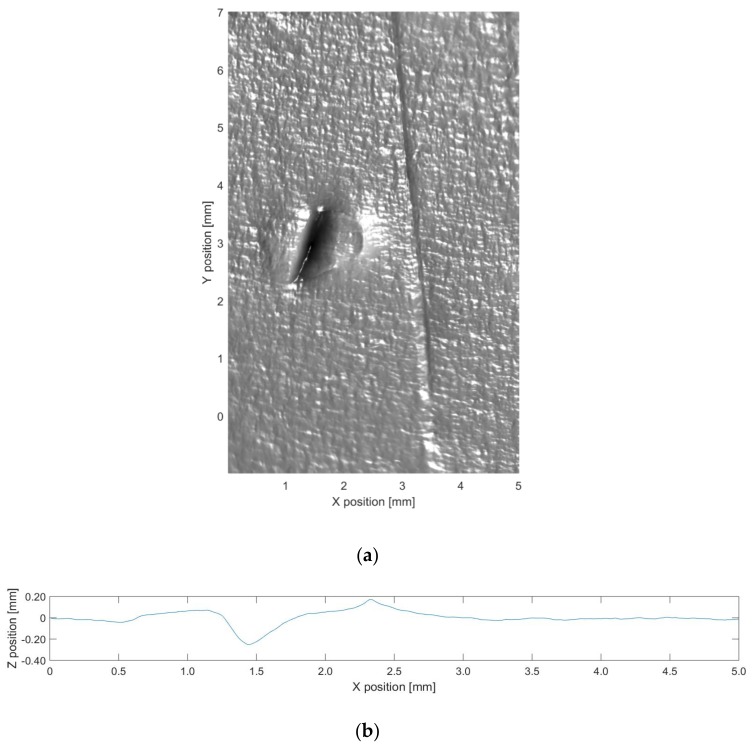
(**a**) Three-dimensional (3D) reconstruction results and (**b**) altimetric readings for the maximum depth in the 3D result for defect 1.

**Figure 6:**
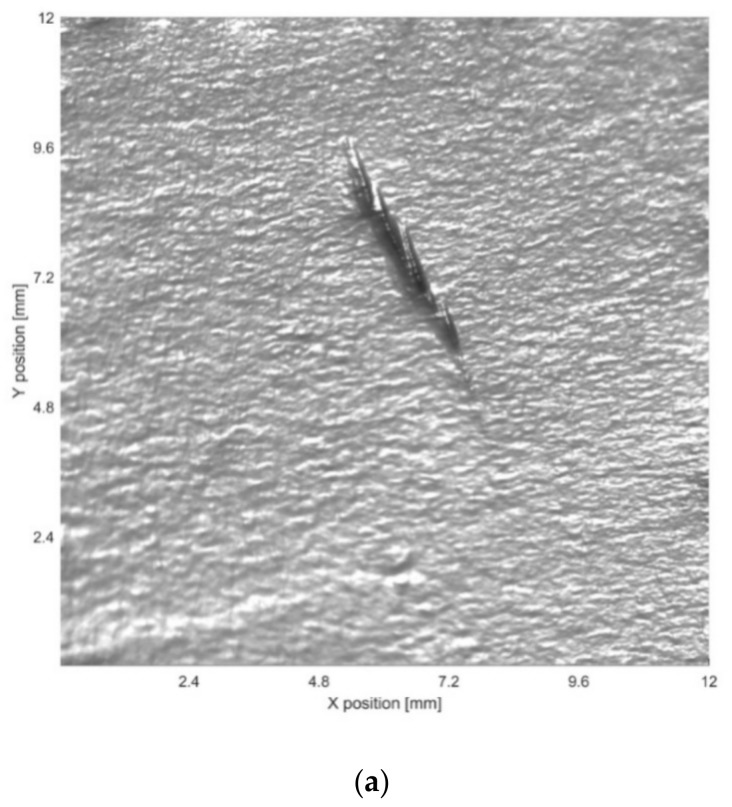

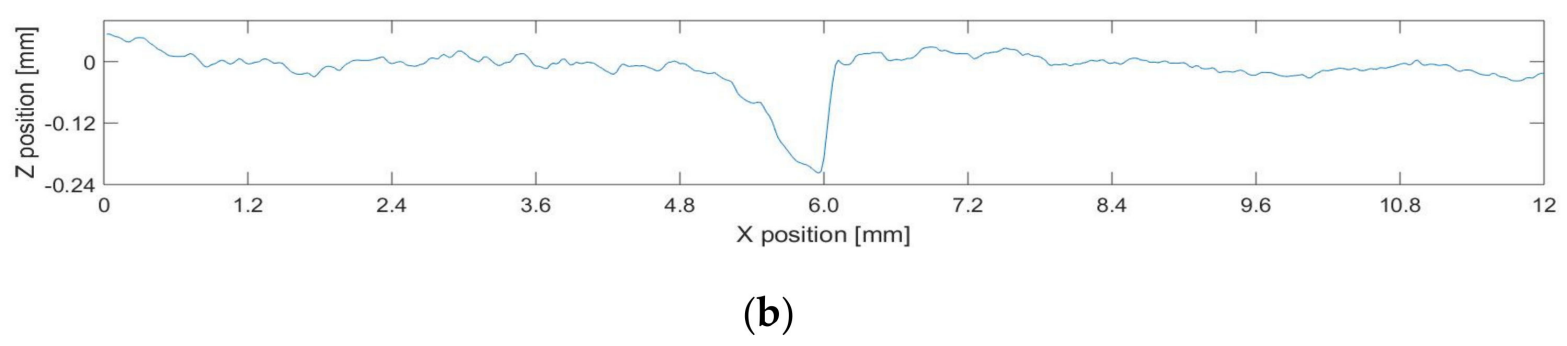
(**a**) 3D reconstruction results and (**b**) altimetric readings for the maximum depth in the 3D result for defect 2.

**Figure 7:**
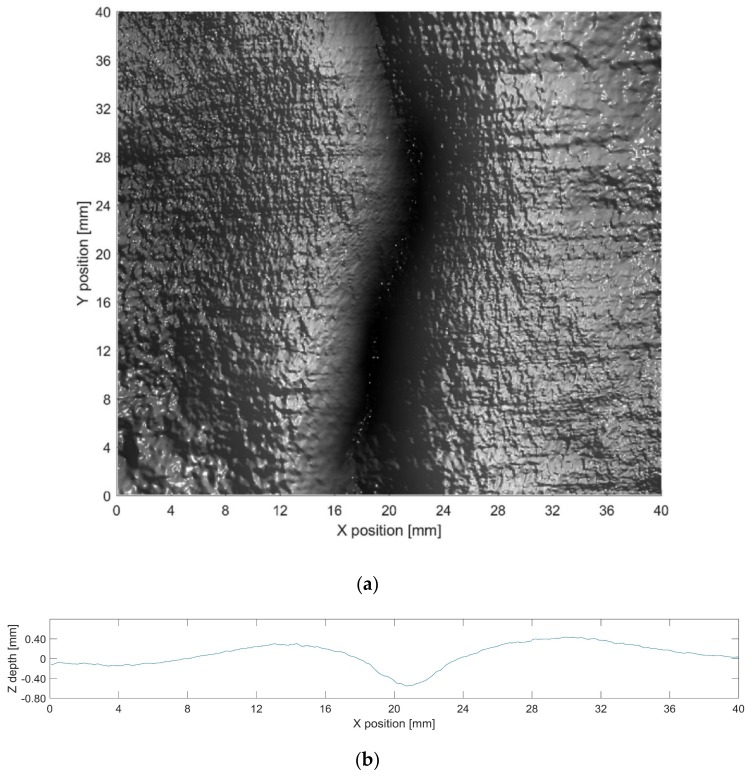
(**a**) 3D reconstruction results and (**b**) altimetric readings for the maximum depth in the 3D result for defect 3.

The 3D data generated for each defect are shown in Figure 5, Figure 6 and Figure 7. The transformation from image pixels to real-world coordinates was achieved as described in Section 3.4. The depth information was then extracted five times along the *Y* direction for each defect, and the maximum depth contour was provided. The 3D data were then combined with altimetric readings of the maximum depth contour for further analysis.

It is clear that the proposed method produces significantly more information than can be observed in the original acquisition images, enabling an accurate characterization of the dimensions and depth of defects. This facilitates image analysis for a range of inspection tasks that could not be solved by 2D analysis of the original acquisition images. 

The initial results of the 3D reconstruction show that the proposed method is very useful for identifying defects, and can contribute to more reliable visual inspections with currently available devices. Thus, exploiting available devices will contribute to significant improvements and time savings for NPP visual inspections.

Table 2 shows the evaluation of defect depth. We compare our method with the baseline derived from MarSurf LD 120, which can conduct measurements using the Mahr metrology products. *Z* is the average maximum depth of the five contour lines extracted from the 3D results. The baseline is the average maximum depth of the five contour lines obtained by LD 120, which uses the non-defect area of the metal plate as the reference. LD 120 measures by contact and has a resolution of 0.001 mm in the depth direction, whereas the capacity for our image sensor and lens is approximately 0.030 mm under the proposed setup. 

As shown in Table 2, the depth estimated by our method is close to the baseline. Errors in the experimental setup come from the camera, light sources, object reflectance, etc. The PS technique can deal with these errors to ensure more precise results; however, all PS techniques rely on radiance measurements. The characteristics of the defects, including defect opening displacement and geometry, will affect the validity of the results. In future research, we will determine the parameters and their impacts on the efficacy of depth measurement, which will require further experiments and verification.

Additionally, there are a few limitations of this study. First, it is assumed that the surface reflectance of the object follows the Lambertian model. Figure 6 reveals no highlights or shadows; therefore, there is no distortion in the results. Conversely, Figure 5 and Figure 7 reveal the distortion in specific areas because of specular reflection. The processing of highlights and shadows requires more images, yet the small number of PTZ lights limits highlight and shadow processing. A methodology to handle shadows and highlights with four lights may be useful to improve the performance [28]. It is also important to note that the experimental defects analyzed in this study are more easily processed than the defects observed under actual inspection conditions. That is, the reflectance of the objects does not deviate substantially from the Lambertian model, and the experiment does not include any underwater images. Thus, the next step is to perform extensive experiments under a range of different conditions. 

The limitations discussed here do not limit the use of this technique in NPP visual inspections. Moreover, we suggest that this research provides an important basis for developing a method that can readily identify and quantify defects through NPP visual inspection.

## 5. Conclusions

This study presents a 3D shape reconstruction method for the visual inspection of defects in NPP reactors. The method is based on the photometric stereo techniques and does not necessitate new inspection devices. The proposed approach, which involves estimating the light source directions and intensities, has reduced the limitation of light calibration and exhibits good practical applicability. The developed methodology can obtain the 3D shape and depth information of defects, thereby improving NPP visual inspection.

The demands for 3D image reconstruction will allow the visual inspection sector to perform more complex and accurate tasks. However, this is only possible if both the software and hardware are improved. The new market applications are expected to continue to emerge as the benefits of a 3D generating function are revealed. This research may also be relevant for designing inspection devices for future generations of NPP reactors.

## Figures and Tables

**Table 1:** Estimation results of the distance between the camera plane and the target plane (mm).

Real Distance	Estimated Value	Error
133	138	5
193	196	3
253	-	-
313	318	5
375	376	1
433	441	8
493	510	17

**Table 2:** Comparison of defect depth calculated from the 3D results and obtained by LD 120 [mm].

Defect Number	*Z*	Baseline
1	0.29	0.23
2	0.22	0.20
3	0.53	0.49
